# Regular Moderate- to Vigorous-Intensity Physical Activity Rather Than Walking Is Associated with Enhanced Cognitive Functions and Mental Health in Young Adults

**DOI:** 10.3390/ijerph17020614

**Published:** 2020-01-18

**Authors:** Takumi Nakagawa, Ibuki Koan, Chong Chen, Toshio Matsubara, Kosuke Hagiwara, Huijie Lei, Masako Hirotsu, Hirotaka Yamagata, Shin Nakagawa

**Affiliations:** Division of Neuropsychiatry, Department of Neuroscience, Yamaguchi University Graduate School of Medicine, Yamaguchi 755-8505, Japan; wyiikura.0904@gmail.com (T.N.); k.ibuki1932@gmail.com (I.K.); t-matsu@yamaguchi-u.ac.jp (T.M.); khagi@yamaguchi-u.ac.jp (K.H.); huijielei@gmail.com (H.L.); hirotsu@yamaguchi-u.ac.jp (M.H.); gata@yamaguchi-u.ac.jp (H.Y.); snakaga@yamaguchi-u.ac.jp (S.N.)

**Keywords:** active coping, cognitive functions, creativity, mental health, moderate- to vigorous-intensity physical activity (MVPA), personal growth, psychological wellbeing, regular physical exercise, state anxiety, working memory

## Abstract

The beneficial effect of physical activity (PA) on the brain has been well established. Both acute and regular PA can boost a range of cognitive functions and enhance mood and mental health. Notably, the effect of acute PA on the brain and cognitive functions is generally found to be dose-dependent, in terms of both the amount and intensity of the exercise episode. In contrast, in the case of regular PA, the literature has primarily focused on the amount of exercise, and limited studies have assessed the influence of the exercise intensity. Since PA in higher intensity causes more extensive, more powerful, and longer-lasting neurobiological changes, it may prove more beneficial to cognitive functions and mental health. In the present study, we set out to test this hypothesis by employing a battery of questionnaires and laboratory tests with a sample of young adults. We found that more frequent vigorous- and moderate-intensity PA rather than walking (considered low to moderate intensity) was associated with better cognitive and mental health measures. Meanwhile, compared with no moderate- to vigorous-intensity physical activity (MVPA) at all, as few as 1~2 days per week (lasting at least 10 min each time) of MVPA was associated with a variety of benefits, particularly related to coping with challenging situations. In light of the neurobiological literature, the present study speaks to the value of moderate- to vigorous- rather than low-intensity PA in enhancing cognitive functions and mental health.

## 1. Introduction

The beneficial effect of physical exercise or physical activity (PA) on the brain has been well established. Both acute and regular PA can boost a range of cognitive functions and enhance mood and mental health [1,2,3,4,5]. For instance, a single bout of aerobic exercise such as treadmill running enhances working memory [6], inhibitory control capacity [7], attentional orienting [8], creativity [9], and positive moods [10]. Regular PA conducted several times a week, such as running and popular sports, can promote cognitive development [11,12], slow cognitive aging [13], buffer stress response [14], and prevent [15] and treat [16] depression.

Furthermore, the beneficial effect of PA is generally considered dose-dependent, such that greater amount and higher intensity of exercise is associated with more enhanced outcomes, for instance, cognitive functions [17]. However, a closer look at the literature suggests this dose-dependent effect in terms of both amount and intensity has only been formally tested in acute but not regular PA (see [17,18] on cognitive functions; see [19] on adult neurogenesis in animals, i.e., mice). In the case of regular PA, the literature has primarily focused on the amount (or frequency and duration) of exercise (see [20] on cognitive functions, i.e., dementia; see [14] on stress resilience; see [15,21,22] on depression or general mental health), and only limited studies have assessed the influence of exercise intensity (see [23,24] on depression).

Since PA performed in higher intensity causes more extensive, more powerful, and longer-lasting neurobiological changes, it may prove more beneficial to brain functions. Indeed, a single bout of vigorous- rather than low-intensity cycling has been reported to increase the peripheral level of brain-derived neurotrophic factor (BDNF) [25]. BDNF is a member of the neurotrophin family of growth factors that support the production, growth, differentiation, and survival of neurons. Peripheral BDNF can pass the brain–blood barrier and benefits the brain through, for instance, enhancing neurogenesis in the dentate gyrus of the hippocampus [5]. Similarly, an episode of moderate to vigorous running but not low-intensity walking increases circulating endocannabinoids [26], which pass the brain–blood barrier, act as a neurotransmitter believed to contribute to “runner’s high”, and have analgesic and anti-anxiolytic effects ([26]; see [27] for a study conducted in mice). Lastly, PA at vigorous intensity is more effective at increasing aerobic capacity or fitness [28], the latter being linked to higher cognitive functions [29] and lower stress response [30] (for a review, see [4]).

Therefore, we hypothesized that frequent PA at moderate and vigorous rather than low intensity exerts greater benefit to cognitive functions and mental health. In the present study, we set out to test this hypothesis in a sample of young adults using a battery of questionnaires and laboratory tests. Investigating this hypothesis also allows us to propose more specific recommendations or guidelines for public health promotion in young adults.

## 2. Materials and Methods

### 2.1. Participants

This research was part of a larger study, the aim of which was to predict mental health status using high-level brain functions. Data collected at baseline of the study were used for analysis here. The study was approved by the Institutional Review Board of Yamaguchi University Hospital (approval code: H2019-043-2). Participants were recruited via numerous posters displayed in the university community. The inclusion criteria were being 20–39 years old at the time of the visit, and the exclusion criteria were (a) having any self-reported mental health diseases, (b) receiving medical examinations due to suspicion of any mental health diseases, (c) being suspected of mental health diseases by the research staff and then diagnosed as having a mental health disease by the Mini-International Neuropsychiatric Interview conducted by a psychiatrist, or (d) being unable to answer the questionnaires and perform the laboratory tests for this study due to severe physical conditions or other reasons. Fifty-eight subjects agreed to participate in this study and provided written informed consent after receiving a detailed explanation of the study.

### 2.2. Measures

#### 2.2.1. Demographic Information

Subjects first answered questions about their demographic characteristics, including gender, age, occupation, and educational level.

#### 2.2.2. PA

To evaluate PA, we used the short-form International Physical Activity Questionnaire (IPAQ) [31]. IPAQ measures the number of days and duration of PA that was conducted for at least ten minutes each time at vigorous intensity, moderate intensity, and walking during the last seven days, respectively. Here, PA at vigorous intensity refers to those that take hard effort to conduct and make people “breathe much harder than normal”. Examples include heavy lifting, fast bicycling, and singles tennis. PA at moderate intensity refers to those that take moderate effort and make people “breathe somewhat harder than normal”. Examples include carrying light loads, swimming at a regular pace, and doubles tennis. Walking is evaluated separately from PA at vigorous and moderate intensity in IPAQ, but is generally considered low to moderate in intensity.

In the present study, we employed two different methods generated by the IPAQ to evaluate PA. First, we categorized the participants into one of three levels of PA, Low, Moderate, or High, based on one summary indicator, total weekly PA Metabolic Equivalents (MET-minutes, hereafter referred as total PA), as suggested by the developers of the IPAQ. Total PA was calculated by weighting the reported minutes per week within each activity category by a MET energy expenditure estimate assigned to each activity category (8 for vigorous, 4 for moderate, and 3.3 for walking). High PA level is defined as meeting either of two criteria: (a) vigorous intensity activity on >3 days/week and accumulating at least 1500 MET-minutes/week; or (b) >7 days of any combination of walking, moderate-intensity, or vigorous-intensity PA achieving at least 3000 MET-minutes/week. Moderate PA level is defined as meeting any of the following three criteria: (a) 3 days of vigorous-intensity activity of at least 20 min/day; (b) 5 days of moderate-intensity activity or walking of >30 min/day; or (c) 5 days of any combination of walking, moderate-intensity or vigorous-intensity PA achieving at least 600 MET-minutes/week. Those who neither meet the Moderate nor the High criteria were categorized as Low PA level.

Second, we employed the intensity-specific frequency, that is, the number of days per week performing activities at each intensity, respectively, in our analysis. Although the first method of evaluating PA (in terms of Low, Moderate, and High PA levels) has been widely used in the literature, it is actually not a very good representative of exercise intensity. Subjects who walk and do moderate PA several days a week and much time each day without doing much vigorous activity can be categorized to High PA level. Therefore, to differentiate the effect of PA at different intensities, we also employed the intensity-specific frequency in our analysis. Another strength of this method is that it allows us to give specific recommendations about the minimum days of intensity-specific PA per week that can bring cognitive and/or mental benefits, the approach of which has been frequently employed in the literature [14,20]. To give the general public specific recommendations, we also combined the frequency of moderate- and vigorous-intensity PA to form a new measure, the frequency of moderate- to vigorous-intensity physical activity (MVPA).

#### 2.2.3. Cognitive Functions

*Creativity*. We used a paper-based quiz composed of insight tasks to evaluate creativity. This included two matchstick arithmetic problems [32], the nine-dot puzzle, and a coin puzzle [33]. Subjects had 5 minutes to complete each task (the two matchstick arithmetic problems were treated as one task). After completing the quiz, subjects were asked whether they had seen any of the tasks. It was found that seven subjects have read the first matchstick arithmetic problem and all but two of the 58 subjects correctly solved this problem. Therefore, the first matchstick arithmetic problem was deleted from the quiz. The total number of solved tasks of the remaining three was used as the creativity score (range 0~3). After excluding subjects that have read any of the remaining tasks, data of 49 subjects were available for the final analysis.

*Working memory*. We used a computer-based n-back task (n = 1, 2) to assess working memory. The task was programed by Jörn Alexander Quent after [34] using MATLAB and Psychtoolbox 3 (the code is available at [35]). In this task, participants were shown a sequence of visual stimuli (random shapes) and had to judge each time whether the current stimulus was identical to the one presented n positions back in the sequence. The shapes were shown in black and presented centrally on a gray background for 500 milliseconds (ms) each, followed by a 2500 ms interstimulus interval. Participants were asked to press a predefined key for targets as fast as they can, and no response was required for non-targets. Participants were tested on 1- and 2-back levels in that order, with each level presented for two consecutive blocks, and one block consisted of 20 + n stimuli and contained 6 targets and 14 + n non-targets each. Following the signal detection theory, a discriminability score indicating the overall performance of subject at discriminating targets from non-targets was calculated for 1- and 2-back separately for each subject [36]. The discriminability score (*d*) and response time (in ms) were used for data analysis.

*Mindful attention*. We used the Japanese version [37] of the Mindful Attention Awareness Scale (MAAS) [38]. The MAAS is a 15-item (1–6 Likert scale) self-report questionnaire assessing individual differences in the frequency of mindful states over time.

#### 2.2.4. Mental Health

Trait measures: the following trait-like dimensions of mental health were evaluated.

*Emotional contagion*. We used the Japanese version [39] of the Emotional Contagion Scale (ECS) [40], a 15-item self-report questionnaire, to assess individual differences in susceptibility to emotional contagion of love, happiness, anger, and sadness.

*Emotion regulation*. We used the Japanese version [41] of the Emotion Regulation Questionnaire (ERQ) [42], a 10-item self-report questionnaire, to assess individual differences in their use of two emotion regulation strategies, reappraisal and suppression. Reappraisal, or cognitive reappraisal, refers to a form of cognitive change that reinterprets a potentially emotion-eliciting situation. Suppression, or expressive suppression, refers to a form of response modulation that inhibits ongoing emotion-expressive behaviors.

*Coping*. We used the Japanese version [43] of the Brief Coping Orientation to Problems Experienced Inventory (COPE) [44], a 28-item self-report questionnaire, to assess individuals’ tendency to employ 14 coping strategies, including active coping, planning, positive reframing, acceptance, humor, religion, using emotional support, using instrumental support, self-distraction, denial, venting, substance use, behavioral disengagement, and self-blame.

*Behavioral inhibition and behavioral activation*. We used the Japanese version [45] of the Behavioral Inhibition System and Behavioral Activation System scales (BIS/BAS) [46], a 24-item self-report questionnaire, to assess four biological personality traits of BIS, drive, fun seeking, and reward responsiveness. The BIS measures individuals’ response to aversive stimuli such as anxiety, fear, and worry. Drive measures the degree to which individuals pursue appetitive goals. Fun seeking measures the tendency to seek new, potentially rewarding experiences. Reward responsiveness measures positive responses to reward or preferred outcomes.

*Trait anxiety*: We used the Y-2 subscale of the Japanese version of the State–Trait Anxiety Inventory (STAI) to assess trait anxiety. Note that the two subscales of the STAI were administered together following *Depression*, see below.

State measures: the following state-like dimensions of mental health were evaluated.

*Depression*. We used the Japanese version [47] of the Beck Depression Inventory-II (BDI-II) [48], a 21-item self-report questionnaire, to assess a range of depressive symptoms that occurred within the past two weeks.

*State anxiety*. We used the Y-1 subscale of the Japanese version [49] of the State–Trait Anxiety Inventory (STAI) [50], a 40-item self-report questionnaire, to assess state and trait anxiety.

*Perceived stress*. We used the Japanese version [51] of the Perceived Stress Scale (PSS) [52], a 10-item self-report questionnaire, to assess perceived psychological stress in the past month.

*Psychological wellbeing*. We used the Japanese version [53] of the Ryff’s Psychological Well-being Inventory [54], an 84-item self-report questionnaire, to assess six domains of psychological wellbeing: autonomy, environmental mastery, personal growth, positive relations with others, purpose in life, and self-acceptance. Autonomy measures self-determination and independence. Environmental mastery measures the sense of mastery and competence in managing one’s environment. Personal growth measures the feeling of continued development. The domain positive relations with others evaluates to what extent one has warm, satisfying, and trusting relationships with others. Purpose in life evaluates to what extent one has goals in life and a sense of direction. Self-acceptance evaluates to what extent one possesses a positive attitude towards the self.

### 2.3. Data Analysis

The statistical analysis was done with IBM SPSS Statistics 25.0 (IBM Corp. in Armonk, NY, USA) and MATLAB R2018b (The MathWorks, Inc., Natick, MA, USA). Pearson correlation analysis was used to evaluate associations between exercise measures (i.e., intensity-specific frequency) and other variables (age, cognitive functions, and mental health). The Chi-square test was used to evaluate whether there was an association between PA level and gender and between PA levels and the two frequencies of MVPA. Linear regression was used to evaluate to what extent exercise measures can predict outcome variables, with or without adjusting for covariates such as gender and age. One-way Analysis of Variance (ANOVA) was used to determine whether there were any differences in outcome variables between subjects at the three PA levels (Low, Moderate, and High), with or without gender and age as covariates (conducted as a general linear model). Bonferroni adjustment was used for post hoc comparison. Student’s *t*-test was used to compare differences between two groups means. The level of statistical significance was set at *p* < 0.05.

## 3. Results

The sample consisted of 24 male and 34 female subjects, with a mean age of 22.4 years (standard deviation (SD) 2.40 years). Fifty-six (98.3%) were undergraduate students, 1 was a graduate student, and 1 was working in a hospital as a healthcare provider. Regarding PA, cognitive functions, and mental health, the score (Mean ± SD) of each measure is shown in Table 1.

Subjects categorized as Low, Moderate, and High PA level numbered 14, 29, and 15, respectively. Subjects conducted 3.64 ± 2.40 days/week of walking, 1.50 ± 1.74 days/week of moderate intensity PA, and 1.26 ± 1.62 days/week of vigorous intensity PA, all of which lasted for at least 10 min each time. As shown in Figure S1, males conducted vigorous intensity PA more often than females (2.00 ± 1.98 vs. 0.74 ± 1.05 days per week, t(32.23) = 2.859, *p* < 0.01). No other significant difference in or association with exercise measures was found for gender and age (see Figures S1 and S2).

### 3.1. PA Level

#### Comparison of Cognitive Functions and Mental Health across Different PA Levels

**Figure 1:**
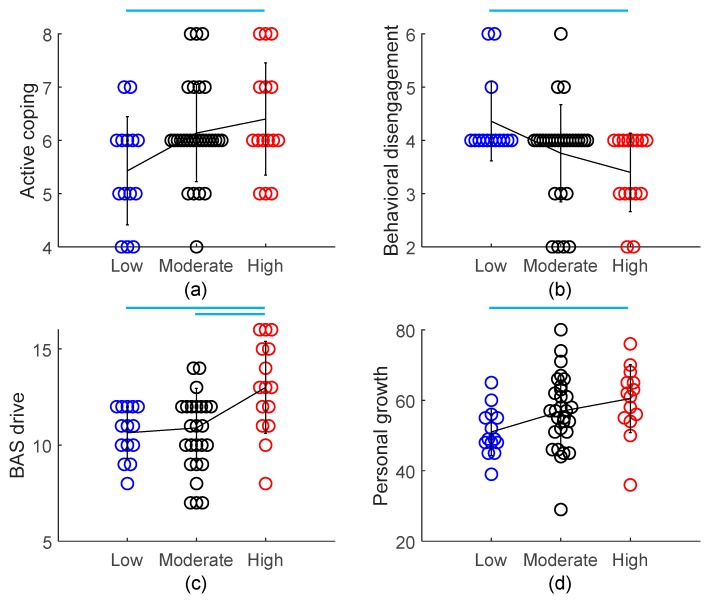
Comparison of active coping (**a**), behavioral disengagement (**b**), BAS drive (**c**), and personal growth (**d**) across different PA levels. n = 14, 29, and 15 for Low, Moderate, and High PA level, respectively. Each circle represents one data point from a single subject. Color indicates different PA levels. The black line connects the three PA levels at their mean value, and the vertical bar drawn on the mean value represents SD of subjects at that PA level. **—** indicates a significant between-group difference (*p* < 0.05) as suggested by post hoc comparisons. PA, physical activity; BAS, Behavioral Activation System.

We first compared the outcome variables across different PA levels using one-way ANOVA and found four significant differences: active coping (F(2,55) = 3.91, *p* = 0.026) and behavioral disengagement (F(2,55) = 4.90, *p* = 0.011) for coping, BAS drive (F(2,55) = 6.66, *p* = 0.003), and personal growth (F(2,55) = 3.71, *p* = 0.031) of psychological wellbeing. As shown in Figure 1, post hoc comparisons suggested that compared with those at Low PA level, subjects at High PA level had a significant higher level of active coping, BAS drive, and personal growth, and low level of behavioral disengagement (*p* < 0.05). Similarly, compared with those at Moderate PA level, subjects at High PA level had a significant higher level of BAS drive (*p* < 0.05). PA level-wise scatter plots of other nonsignificant outcome variables are available in Figure S3.

The group difference for behavioral disengagement (F(4,53) = 2.84, *p* = 0.033 for the model, F = 4.69, *p* = 0.013 for PA levels), BAS drive (F(4,53) = 4.10, *p* = 0.006 for the model, F = 7.74, *p* = 0.001 for PA levels), and personal growth (F(4,53) = 3.97, *p* = 0.007 for the model, F = 4.85, *p* = 0.012 for PA levels) across the three PA levels remained significant after controlling gender and age by fitting a general linear model.

### 3.2. Intensity-Specific Frequency

#### 3.2.1. Correlations among Intensity-Specific Frequencies

We confirmed there was a significant correlation between the frequency of walking and the frequency of vigorous-intensity activity (r = −0.288, *p* < 0.05). More days of walking was associated with fewer days of vigorous-intensity activity per week. Given this correlation, we next entered the frequency of each intensity activity into a linear regression model to determine what intensity activities contribute to the outcome variables while controlling other intensity activities.

#### 3.2.2. Linear Regression: Which Frequency Predicts Outcome Variables?

We fitted linear regression models with only the frequencies of different intensity activities as independent variables (Model 1) and including gender and age as covariates (Model 2). Model 1 could significantly predict six outcome variables, including 2-back *d* of the 2-back working memory task, active coping, denial, and behavioral disengagement of coping, and autonomy and personal growth of psychological wellbeing. Four remained significant after controlling gender and age. The results are shown in Table 2.

Therefore, higher frequency (i.e., more days per week) of vigorous-intensity PA predicts more active coping, less behavioral disengagement, greater autonomy, and increased personal growth. Whereas higher frequency of moderate intensity PA predicts less behavioral disengagement, higher frequency of walking predicts more denial with a trend towards significance. Notably, in predicting personal growth, the standardized coefficient of the frequency of vigorous intensity PA was bigger than that of age (0.404 vs. 0.270). The results of other nonsignificant outcome variables for model 1 and model 2 are shown in Table S1.

#### 3.2.3. Combining the Frequency of Moderate- and Vigorous-Intensity PA: The Frequency of MVPA

To give the general public specific recommendations, we created another measure, that is, the frequency of MVPA. The number of subjects conducting 0 to 7 days of MVPA per week was 13, 7, 9, 6, 9, 6, 5, and 3 in that order.

We first checked the correlation between the frequency of MVPA and outcome variables, and the results are shown in Table 3. As can be seen, as the frequency of MVPA increased, subjects’ performance on the 2-back task became better, they used active coping and acceptance more often and denial and behavioral disengagement less often, showed greater drive and responsiveness to rewarding outcomes, and had fewer state anxiety symptoms and increased personal growth.

As the association between the frequency of MVPA and outcome variables seems linear, we next asked what is the minimum days that subjects have to conduct MVPA per week in order to make a difference in any outcome variables. As the number of subjects conducting 0 to 7 days of MVPA per week was 13, 7, 9, 6, 9, 6, 5, and 3 in that order, we combined subjects conducting 1 and 2 days of MVPA per week to a single group (i.e., 1~2 days of MVPA per week, n = 16) and compared it with subjects conducting 0 days of MVPA per week (n = 13). As shown in Figure S4, the two groups did not differ in their total PA (883.6 ± 929.3 vs. 1770.2 ± 1880.8 MET-minutes/week, t(27) = −0.203, *p* = 0.840). Nor did they differ in their proportion of different PA levels (χ² (2) = 0.842, *p* = 0.656).

On average, subjects conducting 1~2 days of MVPA per week did 0.50 ± 0.63 days of vigorous-intensity PA (45.63 ± 67.13 min/day), 1.06 ± 0.85 days of moderate-intensity PA (66.25 ± 69.37 min/day), and 3.75 ± 1.92 days of walking (59.06 ± 62.96 min/day). In contrast, subjects conducting 0 days of MVPA per week walked 4.77 ± 2.62 days per week (49.46 ± 40.07 min in total).

**Figure 2:**
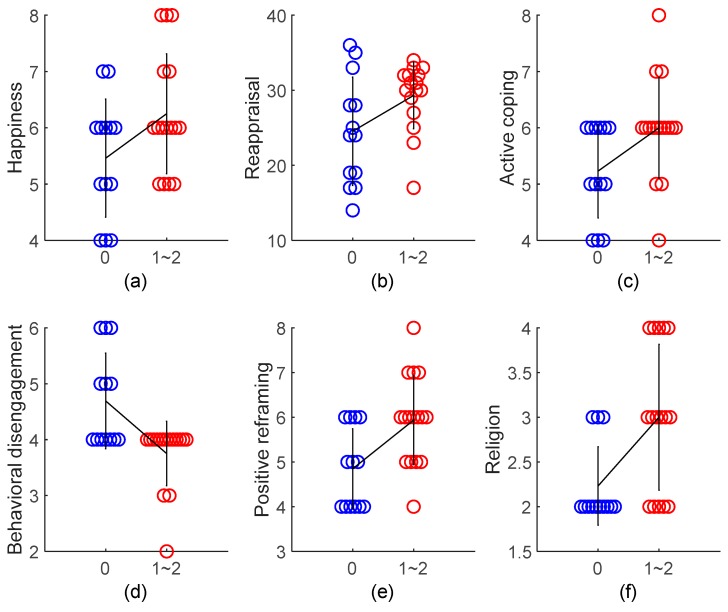
Comparison of happiness (**a**), reappraisal (**b**), active coping (**c**), behavioral disengagement (**d**), positive reframing (**e**), and religion (**f**) between subjects who conducted 0 (n = 13) versus 1~2 days (n = 16) of MVPA per week. Happiness is a submeasure of emotional contagion (ECS); reappraisal is a submeasure of emotion regulation (ERQ); the remaining are submeasures of coping (COPE). Each circle represents one data point from a single subject. Color indicates different groups. The black line connects the two groups at their mean value, and the vertical bar drawn on the mean value represents SD of subjects in that group. Student’s *t*-test, all *p* < 0.05 except happiness, *p* = 0.056 and reappraisal, *p* = 0.050. MVPA, moderate- to vigorous-intensity physical activity.

We then compared the outcome variables of these two groups using Student’s *t*-test. Outcome variables that demonstrated a significant between-group difference (*p* < 0.05) were plotted in Figure 2. As can be seen, compared with those with no MVPA, subjects conducting 1~2 days of MVPA were more easily affected by happiness, more likely to use reappraisal for emotion regulation, more likely to use active coping, positive reframing, and religion, and less likely to use behavioral disengagement for dealing with challenging situations.

## 4. Discussion

In the present study, we employed two different methods to evaluate PA, PA levels and intensity-specific frequency. Our results showed that compared with those at Low PA level, subjects at High PA level used active coping more often and behavioral disengagement less often, and demonstrated greater drive for rewards and more advanced personal growth. Most of these results, except active coping, remained significant after controlling gender and age. These results suggest that a greater amount of PA is associated with more matured psychological coping strategies in the face of negative situations [55,56], enhanced appetitive motivation, which is often compromised in psychiatric disorders [57,58], and superior psychological development and wellbeing [59].

However, the categorization of PA levels based on the proposed criteria is not without its limitations. As we have introduced, High PA level is defined as meeting either of two criteria: (a) vigorous-intensity activity on >3 days/week and accumulating at least 1500 MET-minutes/week; or (b) >7 days of any combination of walking, moderate-intensity, or vigorous-intensity PA achieving at least 3000 MET-minutes/week. From the categorization criteria, we could infer that vigorous-intensity PA in particular may be responsible for the above benefits, although one cannot be sure if that is the case. Individuals may be categorized as High PA level simply due to their greater amount of moderate-intensity PA and walking, with few vigorous-intensity PA. In other words, we cannot tease apart the contribution of PA at different intensities. This limitation is further emphasized by our observation that the frequency of walking is negatively associated with that of vigorous-intensity PA. Therefore, the sensitivity of PA levels in capturing the true amount of PA may have been compromised and confounded by combining the measure of walking with that of vigorous-intensity PA. In order to investigate what intensity PA contributes greater benefits, it is necessary to employ intensity-specific measures.

For this purpose, in the present study, we employed intensity-specific frequencies, that is, days conducting one specific intensity of PA per week. After controlling gender and age, we found it was vigorous-intensity PA that brought various psychological benefits. More frequent vigorous-intensity PA was associated with more frequent use of active coping and fewer use of behavioral disengagement for coping with challenging situations. It was also associated with greater self-perceived autonomy and personal growth. Notably, in predicting personal growth, the contribution of the frequency of vigorous-intensity PA was much bigger than that of age (standardized coefficient 0.404 vs. 0.270). More frequent moderate-intensity PA was also associated with fewer use of behavioral disengagement. In contrast, the frequency of walking was not associated with any of the outcome variables we investigated.

These results confirm our hypothesis and are consistent with the neurobiological literature that moderate- to vigorous- rather than low-intensity PA causes extensive, powerful, and long-lasting physiological changes, which account for the cognitive and psychological enhancing effects of PA [25,26,27]. Therefore, our results go beyond previous reports that regular PA enhances mental health [14,15,21,22] by specifying that it is moderate- to vigorous-intensity PA, rather than low- to moderate-intensity walking, that is responsible for this benefit.

To give the general public specific recommendations on the frequency of PA, we combined the frequency of moderate and vigorous intensity PA and created a new measure, the frequency of MVPA. With MVPA, we observed significant associations with cognitive functions and state anxiety. Thus, individuals with more frequent MVPA performed better on a 2-back working memory task. They could better differentiate targets from non-targets. Meanwhile, they also demonstrated fewer symptoms of state anxiety. These results are in line with a large amount of reports that PA conducted regularly enhances cognitive functions and mental health [11,12,13,14,15,16]. Rather than low- to moderate-intensity walking, our results indicate that it is MVPA that exerts these benefits.

We next compared subjects who conducted 1~2 days per week (lasting at least 10 minutes each time) of MVPA with those with no MVPA to investigate whether this minimum frequency of MVPA can bring any cognitive and/or mental health benefits. Compared with those with no MVPA, we found that subjects conducting 1~2 days of MVPA were more easily affected by happiness, more likely to use reappraisal for emotion regulation, more likely to use active coping, positive reframing, and religion, and less likely to use behavioral disengagement for dealing with challenging situations. In other words, mere 1~2 days of MVPA per week may bring people more positive emotions and more mature coping strategies [55,56,60].

Note that subjects conducting 1~2 days of MVPA did not differ from those conducting no MVPA in terms of their total PA (MET-minutes/week) or proportion of different PA levels. This again supports the argument that rather than the total amount, more intense PA is preferred. In our study, on average, subjects conducting 1~2 days of MVPA did 0.5 days × 45.63 min/day = 22.82 min of vigorous- and 1.06 days × 66.25 min/day = 70.23 min of moderate-intensity PA per week. This amount of PA is below the level recommended by the World Health Organization for adults [61] of doing at least 150 min of moderate-intensity, or 75 min of vigorous-intensity PA per week, or any equivalent combination of the two.

A limitation of our study is the employment of the short- instead of the full-form IPAQ to evaluate PA. Consequently, we could not make differentiation of various PA domains, that is, PA conducted during work versus during transportation, for housework, or for recreation. Previous research suggests that different domains of PA may have distinct effects on mental health [62]. A second limitation of our study is our sample size is rather small and we conducted multiple tests for some forty dependent variables. Given the explorative nature of our study, we did not perform corrections of the alpha level to control the false discovery rate, and this may have increased the number of false positives. Future research with bigger sample size is required to validate our results by further correcting the alpha level based on established procedures.

A third limitation is the cross-sectional design of this study. The cross-sectional design does not allow us to make firm causal inferences on the associations we identified. It is possible that subjects with higher cognitive functions, more mature coping strategies, and better mental health may be more likely to engage in MVPA. Furthermore, our sample was primarily university students, which does not allow us to generalize our findings to older people or people from other settings. Future research should investigate whether our findings hold in a prospective context and other settings and whether interventions with as few as 1~2 days of MVPA cause meaningful cognitive and mental health changes.

## 5. Conclusions

In a sample of young adults, more frequent vigorous- and moderate-intensity PA rather than walking (considered low to moderate intensity) was associated with better cognitive and mental health measures. Meanwhile, compared with no MVPA at all, as few as 1~2 days per week (lasting at least 10 minutes each time) of MVPA was associated with a variety of benefits related particularly to coping with challenging situations. In light of the neurobiological literature, the present study speaks to the value of moderate- to vigorous- rather than low-intensity PA in enhancing cognitive functions and mental health.

## Figures and Tables

**Table 1:** The score of each measure in this study. n = 49 for creativity and n = 58 for all other measures.

Measure	Mean ± SD	Measure	Mean ± SD
**PA**		**Cognitive functions**	
**PA level**	Low = 14, Moderate = 29, High = 15	**Creativity** (Quiz)	1.00 ± 0.82
		**Working memory**	
**Intensity-specific frequency** (days/week)		1-back *d*	2.77 ± 0.34
Walking	3.64 ± 2.40	1-back response time (ms)	668.2 ± 170.0
Moderate	1.50 ± 1.74	2-back *d*	2.55 ± 0.39
Vigorous	1.26 ± 1.62	2-back response time (ms)	800.1 ± 203.2
		**Mindful attention** (MAAS)	44.1 ± 8.98
**Mental health**			
**Emotional contagion** (ECS)		Substance use	2.66 ± 1.12
Love	9.53 ± 1.58	Behavioral disengagement	3.81 ± 0.89
Happiness	5.86 ± 1.34	Self-blame	5.19 ± 1.53
Anger	8.57 ± 1.73	**BIS/BAS**	
Sadness	8.40 ± 1.61	BIS	20.9 ± 4.57
**Emotion regulation** (ERQ)		Drive	11.4 ± 2.20
Reappraisal	27.8 ± 6.47	Fun seeking	12.1 ± 4.52
Suppression	13.2 ± 4.34	Reward responsiveness	16.7 ± 2.35
**Coping** (COPE)		**Depression** (BDI-II)	7.29 ± 6.45
Active coping	6.03 ± 1.03	**State anxiety** (STAI-Y1)	37.2 ± 7.98
Planning	6.12 ± 1.06	**Trait anxiety** (STAI-Y2)	43.3 ± 9.59
Positive reframing	5.50 ± 1.47	**Perceived stress** (PSS)	18.2 ± 4.80
Acceptance	6.24 ± 1.07	**Psychological wellbeing** (PWI)	
Humor	4.31 ± 1.66	Autonomy	49.5 ± 8.33
Religion	2.81 ± 1.10	Environmental mastery	54.7 ± 8.24
Using emotional support	5.64 ± 1.44	Personal growth	56.4 ± 9.89
Using instrumental support	5.97 ± 1.31	Positive relations with others	58.6 ± 9.18
Self-distraction	5.60 ± 1.20	Purpose in life	56.2 ± 10.0
Denial	2.76 ± 1.07	Self-acceptance	51.0 ± 10.8
Venting	5.34 ± 1.37		

SD, standard deviation; PA, physical activity; MAAS, Mindful Attention Awareness Scale; ECS, Emotional Contagion Scale; ERQ, Emotion Regulation Questionnaire; COPE, Coping Orientation to Problems Experienced Inventory; BIS/BAS, Behavioral Inhibition System and Behavioral Activation System scales; BDI-II, Beck Depression Inventory-II; STAI, State–Trait Anxiety Inventory; PSS, Perceived Stress Scale; PWI, Psychological Well-being Inventory.

**Table 2:** Linear regression results using intensity-specific frequencies to predict measures of coping and psychological wellbeing.

	Independent Variables	Working Memory	Coping (COPE)			Psychological Wellbeing (PWI)	
		2-back *d*	Active Coping	Denial	Behavioral Disengagement	Autonomy	Personal Growth
Model 1	**Walking** (days/week)	−0.026	0.001	**0.124** *	−0.006	0.352	0.357
	**Moderate** (days/week)	**0.068** *	0.085	−0.084	**−0.134** * (−0.263) ^1^	−0.616	1.27
	**Vigorous** (days/week)	0.034	**0.251** **	−0.123	**−0.245** ** (−0.447)	**2.08** **	**2.32** **
	F(3,54)	3.122	3.460	3.439	5.409	4.025	3.319
	R^2^	0.148	0.161	0.160	0.231	0.183	0.156
	p	0.033	0.023	0.023	0.003	0.012	0.026
Model 2	**Walking** (days/week)	−0.023	−0.005	**0.118** +	−0.003	0.354	0.226
	**Moderate** (days/week)	**0.071** *	0.087	−0.089	**−0.131** * (−0.257)	−0.627	1.16
	**Vigorous** (days/week)	0.035	**0.298** **	−0.119	**−0.248** ** (−0.452)	**1.885** *	**2.467** ** (0.404)
	**Gender** ^2^	−0.011	0.424	0.079	−0.045	−1.605	2.147
	**Age**	−0.022	0.035	0.051	−0.029	0.061	**1.111** * (0.270)
	F(5,52)	2.089	2.670	2.201	3.247	2.452	3.262
	R^2^	0.167	0.204	0.175	0.238	0.191	0.239
	p	0.082	0.032	0.068	0.013	0.045	0.012

^1^ Unstandardized and standardized coefficients are shown outside of and in the brackets, respectively. ^2^ Male and female are coded as 1 and 2, respectively. ** *p* < 0.01; * *p* < 0.05; + *p* < 0.06. Significant standardized coefficients are shown in bold.

**Table 3:** Pearson correlations between the frequency of MVPA (moderate- to vigorous-intensity physical activity) and outcome variables. n = 49 for creativity and n = 58 for all other measures.

Measure	Correlation Coefficient	Measure	Correlation Coefficient
**Cognitive functions**			
**Creativity** (Quiz)	0.275		
**Working memory**		2-back *d*	**0.343** **
1-back *d*	−0.046	2-back response time	−0.087
1-back response time	−0.101	**Mindful attention** (MAAS)	−0.165
**Mental health**			
**Emotional contagion** (ECS)		Substance use	−0.034
Love	0.199	Behavioral disengagement	−**0.454** **
Happiness	−0.047	Self-blame	0.076
Anger	−0.125	**BIS/BAS**	
Sadness	0.107	BIS	−0.024
**Emotion regulation** (ERQ)		Drive	**0.291** *
Reappraisal	0.159	Fun seeking	−0.027
Suppression	0.013	Reward responsiveness	**0.269** *
**Coping** (COPE)		**Depression** (BDI-II)	0.014
Active coping	**0.345** **	**State anxiety** (STAI-Y1)	−**0.268** *
Planning	0.193	**Trait anxiety** (STAI-Y2)	−0.150
Positive reframing	0.136	**Perceived stress** (PSS)	−0.008
Acceptance	**0.280** *	**Psychological wellbeing** (PWI)	
Humor	−0.099	Autonomy	0.136
Religion	0.162	Environmental mastery	0.189
Using emotional support	0.222	Personal growth	**0.367** *
Using instrumental support	−0.015	Positive relations with others	0.157
Self-distraction	−0.210	Purpose in life	0.236
Denial	**−0.287** *	Self-acceptance	0.204
Venting	−0.228		

** *p* < 0.01; * *p* < 0.05. Significant correlation coefficients are shown in bold.

## Supplementary Materials

The following are available online at https://www.mdpi.com/1660-4601/17/2/614/s1, Figure S1: Gender differences in exercise measures; Figure S2: Associations between age and exercise measures; Figure S3: Comparison of outcome variables across different PA levels; Figure S4: Comparison of total PA and the proportion of different PA levels between subjects conducting 1~2 days of MVPA per week and those conducting no MVPA; Table S1: Additional linear regression results using intensity-specific frequencies to predict cognitive functions and mental health.
