# Supplementary material for: Regular Moderate- to Vigorous-Intensity Physical Activity Rather Than Walking Is Associated with Enhanced Cognitive Functions and Mental Health in Young Adults
Source: Int J Environ Res Public Health. 2020 Jan 18;17(2):614. doi: 10.3390/ijerph17020614 (PMC7014044; doi:10.3390/ijerph17020614)
Supplement: Supplementary file 1 [file ijerph-17-00614-s001.pdf]

International Journal of
Environmental Research and Public Health

MDPI

Supplementary Materials.

Figure S1. Gender differences in exercise measures.

Figure S2. Associations between age and exercise measures.

Figure S3. Comparison of outcome variables across different PA levels.

Table S1. Additional linear regression results using intensity-specific frequencies to predict cognitive functions and mental health.

Figure S4. Comparison of total PA and the proportion of different PA levels between subjects conducting 1~2 days of MVPA per week and those conducting no MVPA.

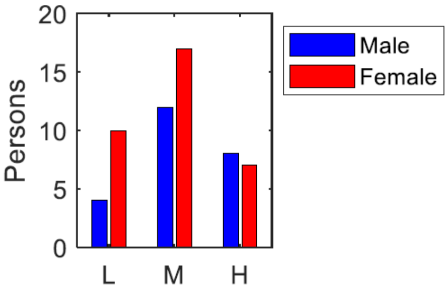

(a)

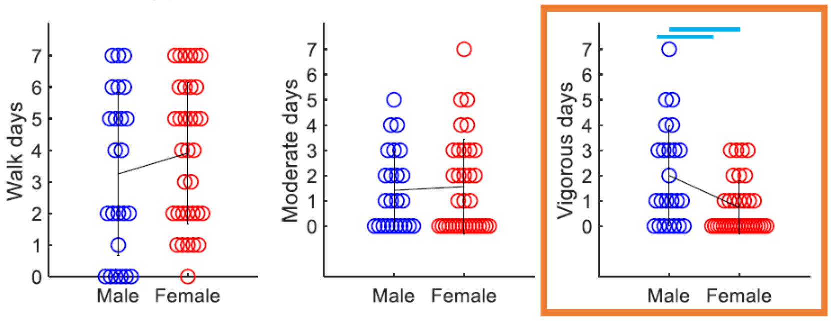

(b) (c) (d)

Figure S1. Gender differences in exercise indices: (a), PA levels, L, M, and H stand for Low, Moderate, and High PA level, respectively (n = 14, 29, and 15 in that order); (b) to (d), intensity-specific frequencies (days/week). For (b) to (d), each circle represents one data point from a single subject. Color indicates different genders. The black line connects the two groups at their mean value and the vertical bar drawn on the mean value represents SD of subjects at that group. — indicates a significant between group difference ($p < 0.05$). As shown in (d), males conducted vigorous intensity PA more often than females (2.00±1.98 vs. 0.74 ±1.05 days per week, t(32.23) = 2.859, $p < 0.01$). None of other comparisons are significant at $p < 0.05$ level. PA, physical activity.

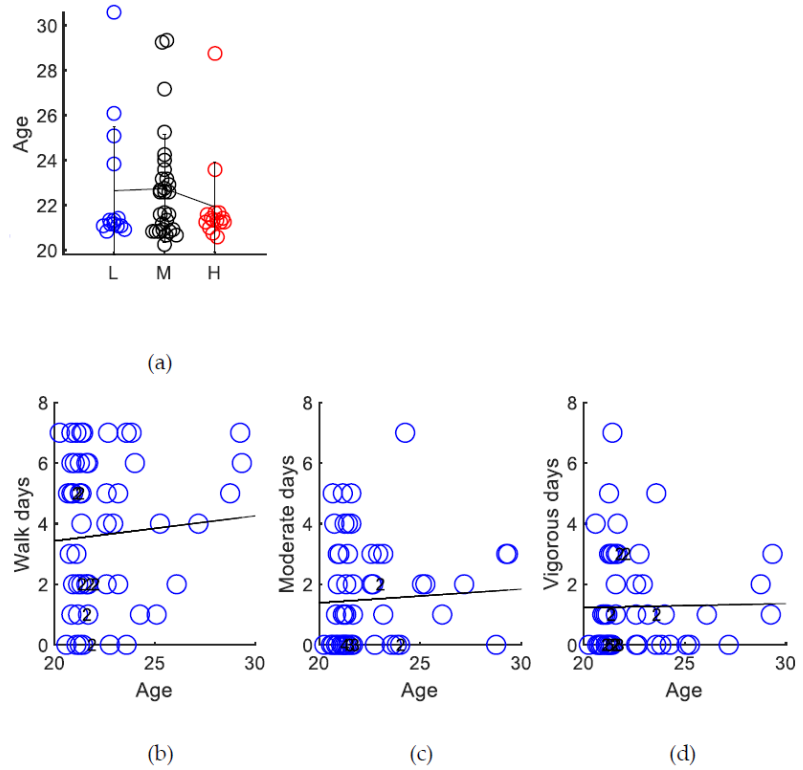

Figure S2. Associations between age and exercise indices: (a), PA levels, L, M, and H stand for Low, Moderate, and High PA level, respectively, which are also indicated by different colors (n = 14, 29, and 15 in that order); (b) to (d), intensity-specific frequencies (days/week). Each circle represents one data point from a single subject. For (a), color indicates different groups, the black line connects the two groups at their mean value and the vertical bar drawn on the mean value represents the standard deviation of subjects at that group. For (b) to (d), the number of subjects with the same value is indicated right to the circle. No correlations or between-group differences are significant at $p < 0.05$ level.

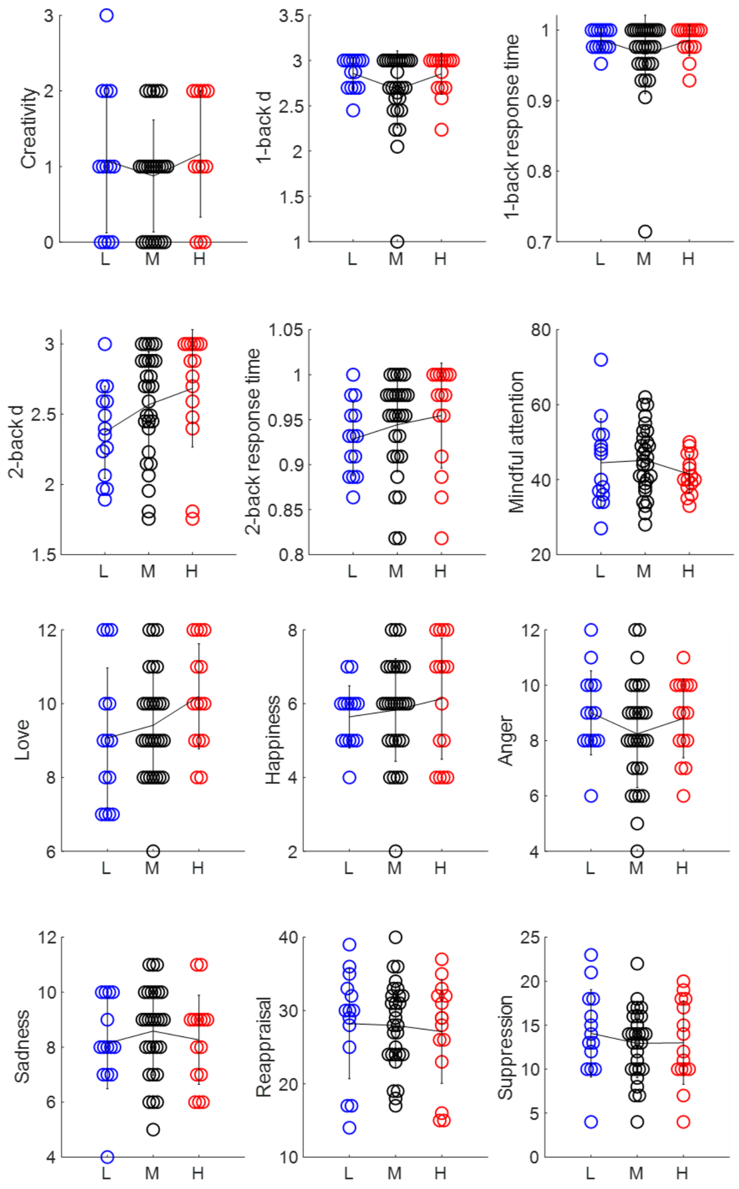
Creativity
1-back d
1-back response time
2-back d
2-back response time
Mindful attention
Love
Happiness
Anger
Sadness
Reappraisal
Suppression
L
M
H

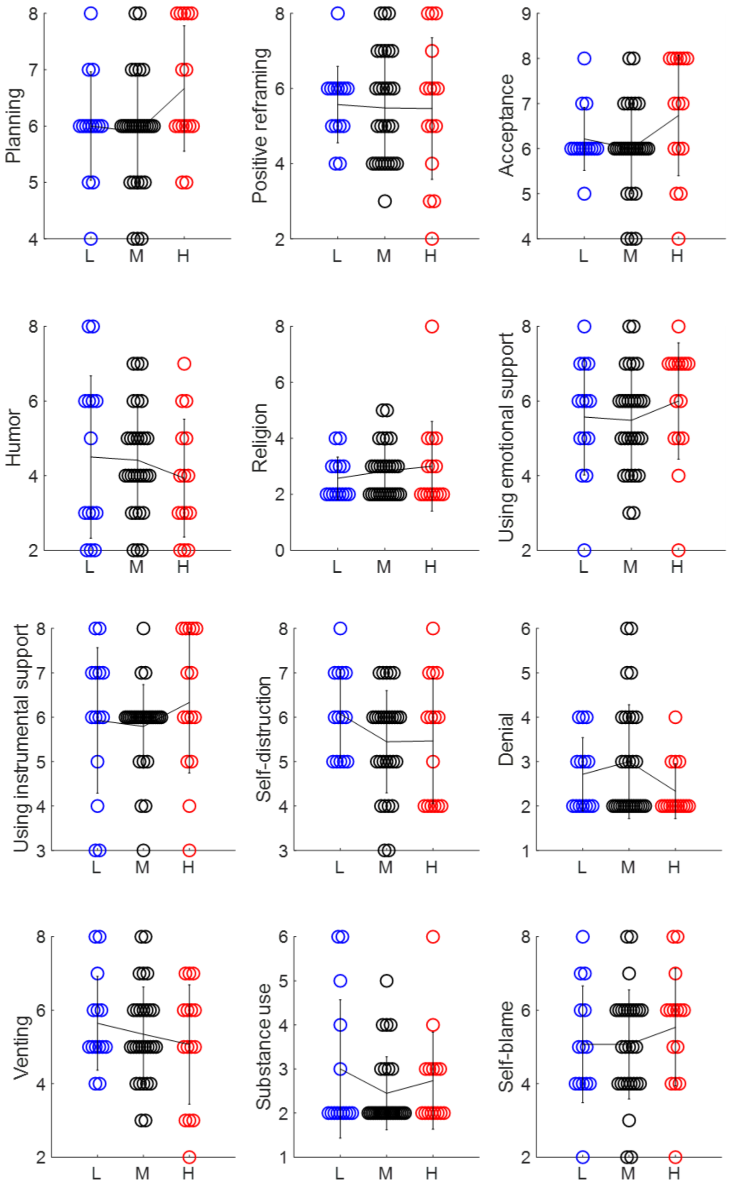
Planning
Positive reframing
Acceptance
Humor
Religion
Using emotional support
Using instrumental support
Self-distruction
Denial
Venting
Substance use
Self-blame
L
M
H

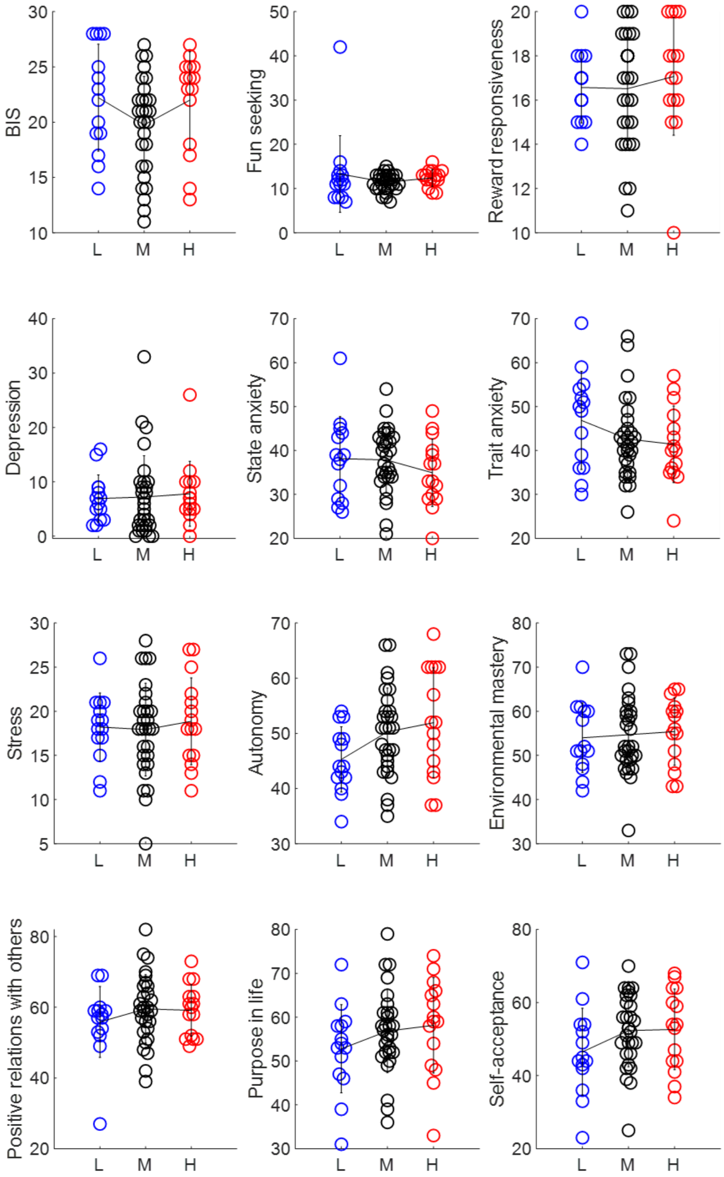

Figure S3. Comparison of outcome variables across different PA levels. L, M, and H stand for Low, Moderate, and High PA level, respectively, which are also indicated by different colors (n = 14, 29, and 15 in that order). Each circle represents one data point from a single subject. The black line connects the three PA levels at their mean value and the vertical bar drawn on the mean value represents SD of subjects at that PA level. None of the between-group differences are significant at $p < 0.05$ level.

Table S1. Additional linear regression results using intensity-specific frequencies to predict cognitive functions and mental health. Notably, after controlling gender and age, vigorous intensity activities and walking could positively and negative predict use of emotional support for coping (standardized coefficient 0.300 vs −0.215), with gender (male and female are coded as 1 and 2, respectively) also being significant (standardized coefficient 0.635). Furthermore, gender could significantly predict emotional contagion of sadness (standardized coefficient 1.321) and positive relations with others (unstandardized coefficient 7.354). ***p* < 0.01; * *p* < 0.05; + *p* < 0.06.

1	Independent variables	Quiz	Working memory		
		**Creativity**	**1-back *d***	**1-back response time**	**2-back response time**
Model 1	**Walking** (days/week)	−0.066	0.003	21.41	16.58
	**Moderate** (days/week)	0.075	−0.006	−17.54	−14.23
	**Vigorous** (days/week)	0.095	−0.006	14.87	10.17
	F	1.892	0.046	2.580	1.088
	R^2	0.112	0.003	0.127	0.057
	p	0.144	0.987	0.063	0.362
Model 2	**Walking** (days/week)	−0.056	0.002	22.05	15.92
	**Moderate** (days/week)	0.072	−0.008	−16.84	−14.60
	**Vigorous** (days/week)	0.041	-0.024	14.25	13.80
	**Gender**	−0.498	−0.140	−9.888	34.38
	**Age**	0.013	0.018	−5.864	4.353
	F	2.037	0.574	1.594	0.733
	R^2	0.192	0.052	0.135	0.066
	p	0.092	0.720	0.179	0.602

2	Independent variables	MAAS	Emotional contagion (ECS)			
		**Mindful attention**	**Love**	**Happiness**	**Anger**	**Sadness**
Model 1	**Walking** (days/week)	0.244	-0.045	0.131	−0.132	0.013
	**Moderate** (days/week)	−0.163	0.050	0.010	−0.260	0.200
	**Vigorous** (days/week)	−1.119	0.223	0.011	0.008	−0.058
	F	0.986	1.267	1.005	1.909	1.023
	R^2	0.052	0.066	0.053	0.096	0.054
	p	0.406	0.295	0.398	0.139	0.390
Model 2	**Walking** (days/week)	0.175	−0.050	0.115	−0.136	−0.007
	**Moderate** (days/week)	−0.266	0.048	0.000	−0.262	0.191

	**Vigorous** (days/week)	−1.629	0.260	0.094	0.021	0.087
	**Gender**	−3.687	0.340	0.793	0.127	**1.321****
	**Age**	0.830	0.031	0.110	0.025	0.118
	F	1.603	0.879	2.081	1.133	3.082
	R^2	0.134	0.078	0.167	0.098	0.229
	p	0.176	0.502	0.083	0.355	**0.016**

3	Independent variables	Emotion regulation (ERQ)		Coping (COPE)		
		**Reappraisal**	**Suppression**	**Planning**	**Positive reframing**	**Acceptance**
Model 1	**Walking** (days/week)	−0.308	−0.078	0.022	−0.108	-0.039
	**Moderate** (days/week)	0.283	−0.235	−0.016	0.037	0.072
	**Vigorous** (days/week)	0.487	0.284	0.236	0.083	0.184
	F	0.758	0.495	2.583	0.973	2.152
	R^2	0.040	0.027	0.125	0.051	0.107
	p	0.523	0.688	0.063	0.412	0.104
Model 2	**Walking** (days/week)	−0.373	−0.097	0.026	−0.123	−0.037
	**Moderate** (days/week)	0.208	−0.262	−0.012	0.021	0.075
	**Vigorous** (days/week)	0.321	0.158	0.239	0.062	0.190
	**Gender**	−0.895	−0.899	-0.002	−0.068	0.029
	**Age**	0.656	0.219	−0.033	0.141	−0.026
	F	1.183	0.542	1.566	1.208	1.290
	R^2	0.102	0.050	0.131	0.104	0.110
	p	0.330	0.743	0.186	0.318	0.282

4	Independent variables	Coping (COPE)				
		**Humor**	**Religion**	**Using emotional support**	**Using instrumental support**	**Self-distraction**
Model 1	**Walking** (days/week)	0.150	0.051	−0.106	0.022	−0.017
	**Moderate** (days/week)	−0.034	0.046	0.156	-0.024	−0.023
	**Vigorous** (days/week)	−0.026	0.155	0.062	0.023	−0.232
	F	1.015	0.932	1.581	0.058	1.810
	R^2	0.053	0.049	0.081	0.003	0.091
	p	0.393	0.432	0.205	0.981	0.156
Model 2	**Walking** (days/week)	0.137	0.044	**−0.128**+ (−0.215)	0.011	−0.030

	**Moderate** (days/week)	−0.049	0.040	0.149	−0.026	−0.030
	**Vigorous** (days/week)	−0.054	0.164	**0.267*** (0.300)	0.140	−0.147
	**Gender**	−0.132	0.129	**1.835**** (0.635)	1.034	0.789
	**Age**	0.135	0.063	0.114	0.050	0.079
	F	1.057	0.794	9.028	1.742	2.761
	R^2	0.092	0.071	0.465	0.143	0.210
	p	0.395	0.559	**0.000**	0.141	0.028

5	Independent variables	Coping (COPE)			BIS/BAS	
		**Venting**	**Substance use**	**Self-blame**	**BIS**	**Drive**
Model 1	**Walking** (days/week)	0.009	−0.046	0.076	−0.098	0.037
	**Moderate** (days/week)	−0.054	−0.007	0.063	0.132	0.188
	**Vigorous** (days/week)	−0.240	−0.060	0.090	−0.330	0.435
	F	1.618	0.221	0.343	0.319	2.101
	R^2	0.082	0.012	0.019	0.017	0.105
	p	0.196	0.881	0.794	0.812	0.111
Model 2	**Walking** (days/week)	0.014	−0.054	0.069	−0.104	0.031
	**Moderate** (days/week)	−0.042	−0.017	0.057	0.143	0.192
	**Vigorous** (days/week)	−0.142	−0.107	0.096	−0.088	0.575
	**Gender**	0.768	−0.338	0.102	2.027	1.184
	**Age**	−0.083	0.084	0.062	−0.046	−0.005
	F	2.083	0.692	0.313	0.652	2.058
	R^2	0.167	0.062	0.029	0.059	0.165
	p	0.082	0.632	0.903	0.662	0.086

6	Independent variables	BIS/BAS		BDI-II	STAI	
		**Fun seeking**	**Reward responsiveness**	**Depression**	**State anxiety**	**Trait anxiety**
Model 1	**Walking** (days/week)	−0.115	−0.187	0.178	0.199	0.027
	**Moderate** (days/week)	−0.196	0.238	0.012	−0.920	−0.516
	**Vigorous** (days/week)	0.037	0.224	0.189	−0.903	−0.797
	F	0.175	2.128	0.086	1.462	0.441
	R^2	0.010	0.106	0.005	0.075	0.024

	p	0.913	0.107	0.967	0.235	0.725
Model 2	**Walking** (days/week)	−0.113	-0.206	0.129	0.167	−0.014
	**Moderate** (days/week)	−0.182	0.225	−0.019	−0.972	−0.549
	**Vigorous** (days/week)	0.194	0.301	0.424	−1.203	−0.720
	**Gender**	1.270	0.767	2.274	−2.232	0.915
	**Age**	−0.087	0.142	0.344	0.416	0.332
	F	0.299	1.833	0.526	1.235	0.353
	R^2	0.028	0.150	0.048	0.106	0.033
	p	0.911	0.122	0.756	0.306	0.878

7	Independent variables	PSS	Psychological wellbeing (PWI)			
		**Perceived stress**	**Environmental mastery**	**Positive relations with others**	**Purpose in life**	**Self-acceptance**
Model 1	**Walking** (days/week)	0.033	0.033	-0.323	0.054	-0.436
	**Moderate** (days/week)	-0.169	0.931	0.821	1.029	0.386
	**Vigorous** (days/week)	0.185	0.465	0.246	1.158	1.448
	F	0.156	0.773	0.660	1.066	1.341
	R^2	0.009	0.041	0.035	0.056	0.069
	p	0.925	0.514	0.580	0.371	0.271
Model 2	**Walking** (days/week)	0.005	−0.014	−0.441	−0.002	−0.430
	**Moderate** (days/week)	−0.186	0.901	0.764	1.003	0.367
	**Vigorous** (days/week)	0.324	0.676	1.043	1.540	1.105
	**Gender**	1.325	2.055	**7.354****	3.522	−2.856
	**Age**	0.188	0.331	0.728	0.345	0.087
	F	0.365	0.711	2.750	1.019	0.956
	R^2	0.034	0.064	0.209	0.089	0.084
	P	0.870	0.618	**0.028**	0.416	0.435

MAAS, Mindful Attention Awareness Scale; ECS, Emotional Contagion Scale; ERQ, Emotion Regulation Questionnaire; COPE, Coping Orientation to Problems Experienced Inventory; BIS/BAS, Behavioral Inhibition System and Behavioral Activation System scales; BDI-II, Beck Depression Inventory-II; STAI, State–Trait Anxiety Inventory; PSS, Perceived Stress Scale; PWI, Psychological Well-being Inventory.

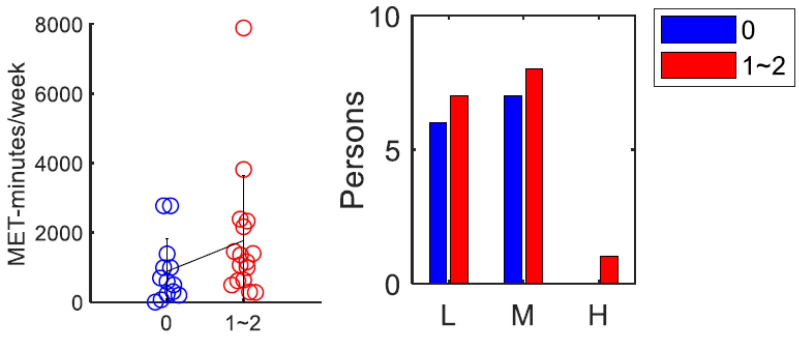

(a) (b)

Figure S4. Comparison of total PA (a) and the proportion of different PA levels (b) between subjects conducting 1~2 days of MVPA per week and those conducting no MVPA. For (a), each circle represents one data point from a single subject. Color indicates different groups. The black line connects the two groups at their mean value and the vertical bar drawn on the mean value represents SD of subjects at that group. Neither of the comparison is significant at $p < 0.05$. MVPA, moderate- to vigorous-intensity physical activity.
